# Reconstruction and analysis of carbon metabolic pathway of *Ketogulonicigenium vulgare* SPU B805 by genome and transcriptome

**DOI:** 10.1038/s41598-018-36038-3

**Published:** 2018-12-13

**Authors:** Cai-Yun Wang, Ye Li, Zi-Wei Gao, Li-Cheng Liu, Ying-Cai Wu, Meng-Yue Zhang, Tian-Yuan Zhang, Yi-Xuan Zhang

**Affiliations:** 1 School of Life Science and Biopharmaceutics, Shenyang Pharmaceutical University, Shenyang, 110016 China; 2 Northeast Pharmaceutical Group Co., Ltd, Shenyang, 110026 People’s Republic of China; 3 Department of Biotechnology, School of Engineering, Nagoya University, Furo-cho, Chikusa-ku, Nagoya, 464-8603 Japan

## Abstract

*Ketogulonicigenium vulgare* has been widely used in vitamin C two-step fermentation. Four *K*. *vulgare* strains (WSH-001, Y25, Hbe602 and SKV) have been completely genome-sequenced, however, less attention was paid to elucidate the reason for the differences in 2-KGA yield on genetic level. Here, a novel *K*. *vulgare* SPU B805 with higher 2-keto-L-gulonic acid (2-KGA) yield, was genome-sequenced to confirm harboring one circular chromosome with plasmid free. Comparative genome analyses showed that the absence of plasmid 2 was an important factor for its high 2-KGA productivity. The amino acid biosynthetic pathways in strain SPU B805 are much more complete than those in other *K*. *vulgare* strains. Meanwhile, strain SPU B805 harbored a complete PPP and TCA route, as well as a disabled EMP and ED pathway, same as to strain SKV, whereas strain WSH-001, Y25 and Hbe602 harbored complete PPP, ED, TCA pathway and a nonfunctional EMP pathway. The transcriptome of strain SPU B805 validated the carbon metabolism in cytoplasm mainly through the PPP pathway due to its higher transcriptional levels. This is the first time to elucidate the underlying mechanism for the difference in 2-KGA yield, and it is of great significance for strain improvement in the industrial fermentation.

## Introduction

*Ketogulonicigenium vulgare* has been widely used for the bioconversion of L-sorbose to 2-keto-L-gulonic acid (2-KGA), the key precursor of vitamin C in the industrial production. In this process, *K*. *vulgare* is usually co-cultured with companion strain, *Bacillus megatherium*, which is generally considered to provide nutrients or metabolic substances for the growth of *K*. *vulgare*^1^. The known 2-KGA-producing strains, *K*. *vulgare* Y25, *K*. *vulgare* WSH-001, *K*. *vulgare* SKV and *K*. *vulgare* Hbe602 have been completely genome-sequenced and reported^2–5^. The genome-scale metabolic model of *K*. *vulgare* WSH-001 was reconstructed and analyzed by flux balance analysis (FBA)^6^. Total 116 genes, located on the chromosome, were identified as the essential genes for the growth of *K*. *vulgare* WSH-001^6^. Among them, approximately half of the essential genes participated in the metabolism of nucleotides and amino acids. Some specific amino acids significantly affected the growth of cells and the accumulation of 2-KGA^7,8^; moreover, the uptake rates of amino acids had a linear positive correlation with cell growth rate^6^. Additional, *K*. *vulgare* WSH-001 had a powerful transporting system to absorb nutrients and various amino acids^3^. Although many researches on metabolic pathways have been carried out, the understanding of the *K*. *vulgare* is still a partial picture due to its unique growth characters. Therefore, it is necessary to discover the panorama of whole metabolism features of different 2-KGA-producing strains.

In prokaryotes, sugar has a variety of oxidation decomposition pathways, the most common of which are the Embden-Meyerhoff-Parnass (EMP) pathway, the Entner-Doudoroff (ED) pathway and the pentose phosphate pathway (PPP)^9^. However, these routes are different in the ATP yield, cofactors and chemical intermediates. The EMP pathway is a process by which glucose is not completely oxidized to pyruvate under anaerobic conditions^10^. In the classical EMP pathway, glucose is phosphorylated twice and cleaved into glyceraldehyde 3-phosphate (G3P) and dihydroxyacetone phosphate (DHAP), and then two G3P are formed by the reversible interconversion between DHAP and G3P. G3P is metabolized to pyruvate in the subsequent glycolysis and coupled with ATP production through the substrate-level phosphorylation. So, one mole of glucose is oxidized to produce a net of two moles of ATP and two moles of NADH through the EMP pathway.

The ED pathway is a wildly distributed glucose catabolic route in nature^11,12^. In the ED pathway, glucose is phosphorylated to form glucose 6-phosphate (G6P), and then oxidized to 6-phosphogluconate (6PG) by glucose-6-phosphate dehydrogenase (Zwf)^11^. 6PG is then oxidized to 2-keto-3-deoxy-6-phosphogluconic acid (KDPG) by KDPG aldolase (Eda), which is then cleaved to one pyruvate and one G3P. G3P is used to produce ATP through substrate-level phosphorylation in latter glycolysis. KDPG is exclusively generated in the ED pathway and Eda is the key enzyme unique to the ED pathway^10^. The ED pathway can achieve the same conversion rate as the EMP pathway do, but fewer enzymes are required^10^. During the process, only one mole of ATP as well as one mole of NADH and one mole of NADPH are produced when one mole of glucose is decomposed.

The PPP pathway, as a fundamental component in the carbon metabolism, can be divided into the oxidative branch and non-oxidative branch^13^. In the oxidative branch, G6P is metabolized into ribulose 5-phosphate (Ru5P), NADPH and carbon dioxide by the enzyme Zwf and 6-phosphate gluconate dehydrogenase (Gnd). In the non-oxidative branch, the glycolytic intermediates fructose 6-phosphate (F6P), G3P and sedoheptulose 7-phosphate (S7P) are rearranged to generate ribose 5-phosphate (R5P) and other sugar phosphate for the subsequent biosynthesis of nucleic acids and amino acids^13^. Zwf plays an important role in the fine-tuning of PPP pathway, but it is not the unique to the PPP pathway because of its product 6PG can be channeled into ED pathway for further metabolism^11^. Unlike the EMP and ED pathway, one mole of glucose is decomposed to produce a net of two moles of NADPH through the PPP pathway.

Although several strains of *K*. *vulgare* have been genome-sequenced, annotated and published^2–5^, but their attentions were mainly focused on the elucidation of the metabolic pathway of 2-KGA biosynthesis and symbiosis mechanism with companion strain^4,14^. Less attention was paid to explore the mechanism of different 2-KGA yield on the genetic level in different strains. Therefore, in this work we studied the differences in genome and carbon metabolism pathway between *K*. *vulgare* SPU B805 and the four reported *K*. *vulgare* strains to understand the variations in the growth and 2-KGA production, as well as the advantages of *K*. *vulgare* SPU B805. *K*. *vulgare* SPU B805, a new 2-KGA-producing strain, contains only a circular chromosome without any plasmid. The COG and RAST annotation revealed that the genes encoding gluconate 2-dehydrogenase (GA2DH), one of L-sorbosone dehydrogenase (SNDH) and the key enzymes (Eda and Edd) unique to the ED pathway were all absent from the genome of strain SPU B805, which may be the underling mechanism that contributes to its high 2-KGA productivity. The complete pathways of glycine, serine, threonine and proline in SPU B805, whereas absent from other *K*. *vulgare* strains, may be another reason for high 2-KGA yield of SPU B805. Meanwhile, the comparative genome analysis showed that strain SPU B805 and SKV contained a complete PPP and TCA cycle, as well as a disabled EMP and ED pathway. Whereas, strain WSH-001, Y25 and Hbe602 harbor complete PPP, ED, TCA pathways and a nonfunctional EMP pathway. The transcriptome of strain SPU B805 validated that the PPP pathway was of major importance for cytoplasmic carbon oxidation. Our findings provide a complementary platform for understanding the underlying mechanism for the different central carbon metabolic features and 2-KGA yield of different *K*. *vulgare* strains. It also provides a useful framework for further physiology and gene engineering research. This is of great significance for *K*. *vulgare* strain improvement and genetic engineering in the 2-KGA industrial fermentation.

## Results

### 2-KGA yield of *K*. *vulgare* SPU B805 in the co-culture system

*K*. *vulgare* SPU B805 was fermented with *B*. *megatherium* SPU B806 in 5-L bioreactor for 2-KGA production. The 2-KGA production was 82.74 ± 0.77 g/L with a 95.97% conversion rate at the end of the fermentation, and the absorbance at 660 nm was 5.63 ± 0.17, which means the cell concentration is 52 ± 1.4 × 10^8^ CFU/mL (Table 1; Supplementary Fig. S1). Because no fermentation data of Y25 and SKV, the 2-KGA production abilities were compared between Hbe602, WSH-001 and SPU B805. As shown in Table 1, strain SPU B805 showed significantly differences (*p* < 0.001) compared with *K*. *vulgare* Hbe602 in the 2-KGA yield (increased by 25.55%, increment of 16.84 mg/ml), productivity (increased by 241.82%), 2-KGA conversion rate (increased by 25.55%) and fermentation cycle (decreased by 172.73%); similarly, strain SPU B805 showed significantly differences (*p* < 0.001) compared with *K*. *vulgare* WSH-001 in the 2-KGA yield (increased by 23.81%, increment of 15.91 mg/ml.), productivity (increased by 102.15%), 2-KGA conversion rate (increased by 8.48%) and fermentation cycle (decreased by 59.09%). The cell growth (OD_660_) of SPU B805 was also significantly higher than that of WSH-001 (*p* < 0.05). These superior qualities aroused our great interest to understand the mechanism. So, the genome of *K*. *vulgare* SPU B805 was sequenced, annotated and compared with all of the reported 2-KGA-producing strains (SKV, Hbe602, WSH-001 and Y25).

**Table 1:** The fermentation parameters of different *K*. *vulgare* strains.

Strain	*K*. *vulgare* SPU B805	*K*. *vulgare* Hbe602	*K*. *vulgare* WSH-001
Cell growth (CFU/mL)	52 ± 1.4 × 10^8^	Null	Null
Cell growth (OD660)	5.63 ± 0.17	Null	5.13 ± 0.12*
Volume (L)	5	5	7
Total L-sorbose (g/L)	80	80	70
Fermentation period (h)	44	120***	70***
2-KGA production (g/L)	82.74 ± 0.77	65.9 ± 0.4***	66.83 ± 1.1***
2-KGA productivity (g/L/h)	1.88 ± 0.02	0.55 ± 0.00***	0.93 ± 0.01***
Conversion rate (mol/mol)	95.97%	76.44%***	88.47%***
References	This study	^14^	^7^

Note: ^*,***^ indicate statistically significant difference when compared with *K*. *vulgare* SPU B805 (*p* < 0.5, 0.001).

### Genomic features of *K*. *vulgare* SPU B805

The complete genome of *K*. *vulgare* SPU B805 was sequenced and deposited in the GenBank database with the accession number CP017622. The genome schema was drawn as Supplementary Fig. S2. It is 3,032,608 bp in length with a G + C content of 61.7%. Based on 16S rRNA gene phylogenetic analysis, *K*. *vulgare* SPU B805 is highly homologous to a reported 2-KGA-producing strain of *K*. *vulgare* SKV^5^ (see Supplementary Fig. S3). The comparison of *K*. *vulgare* SPU B805 and *K*. *vulgare* Y25^2^, *K*. *vulgare* WSH-001^3^, *K*. *vulgare* SKV^5^ and *K*. *vulgare* Hbe602^4^ in genome characteristics was summarized in Table 2. *K*. *vulgare* SPU B805 has the largest genome among all the strains, but it doesn’t encode the most genes and proteins. No plasmid is found in *K*. *vulgare* SPU B805, while other four strains have 1–2 plasmids. The number of rRNA (15) and tRNA (58) are the same as those in other strains except for tRNA (59) in WSH-001. The number of sorbose dehydrogenase (*sdh*) and sorbosone dehydrogenase (*sndh*), key genes responsible for the bioconversion of L-sorbose to 2-KGA, is different among all the strains, shown in Table 2. There are 5 *sdh* genes (KVC_2764, KVC_2744, KVC_0718, KVC_1927, KVC_0337) and 1 *sndh* gene (KVC_0605) in strain SPU B805.

**Table 2:** Genome features of different 2-KGA-producing strains of *K*. *vulgare*.

Strain	*K. vulgare* SPU B805	*K. vulgare* SKV	*K. vulgare* Hbe602	*K. vulgare* WSH-001	*K. vulgare* Y25
chromosome (Mb)	3.03	2.76	2.77	2.77	2.78
G + C (%)	61.7	61.7	61.7	61.7	61.7
Plasmid 1 (bp)	Null	267,949	267,988	267,986	268,675
Plasmid 2 (bp)	Null	Null	242,716	242,715	243,645
Protein	2947	2851	3178	3054	2905
Gene	3062	3067	3281	3198	3286
rRNA	15	15	15	15	15
tRNA	58	58	58	59	58
SDH	5	6	6	5	5
SNDH	1	1	2	2	2
GA2DH	Null	Null	1	1	1
Reference	This work	^5^	^4^	^3^	^2^
Accession number	CP017622	CP016592	CP012908	CP002018	CP002224
			CP012909	CP002019	CP002225
		CP016593	CP012910	CP002020	CP002226

**Figure 1:**
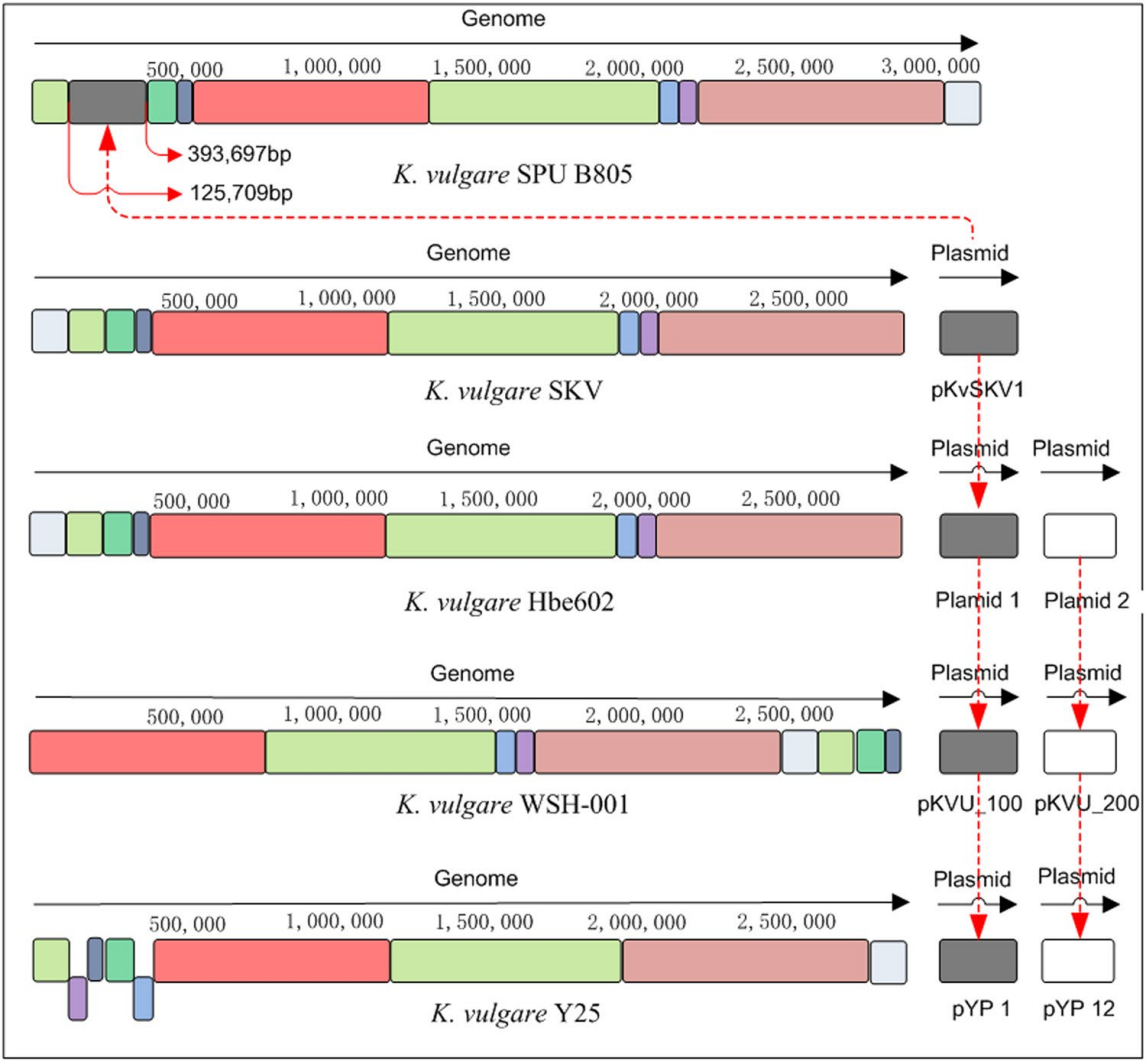
The genome comparison of *K*. *vulgare* SPU B805 with the published genomes. The frames of the same color represent the homologous genome fragments. The frames under the main body represent the inverted fragments. The sequence of plasmid pKvSKV1 (belong to SKV), plasmid 1 (belong to Hbe602), pKVU_100 (belong to WSH-001) and pYP1 (belong to Y25) with highly similarity is inserted into the genome of *K*. *vulgare* SPU B805 (dark grey parts), while plasmid 2 (belong to SKV) or pKVU_200 (belong to WSH-001) or pYP12 (belong to Y25) (white fragments) is missing in the *K*. *vulgare* SPU B805 genome.

Furthermore, the genome-scale sequence comparison conducted by LAST software showed that the genome of *K*. *vulgare* SPU B805 is more similar with *K*. *vulgare* SKV (see Supplementary Fig. S4). Strain SPU B805, showing a 99% identity to strain SKV, harbors one circular chromosome without any plasmid. However, strain SKV contains one circle chromosome and one circle plasmid, while the other strains (Y25, WSH-001, Hbe602) consist of one circle chromosome and two circle plasmids, respectively. Therefore, it is the first time to find a 2-KGA-producing strain without any plasmid. The frame diagram of genome comparison between *K*. *vulgare* SPU B805, SKV, Hbe602, WSH-001 and Y25 was shown in Fig. 1. Most of the genome sequences are highly consistent except for some insertion, dislocation and rearrangement. Further analysis revealed that the DNA fragment of plasmid 1 (for example, namely as plasmid pKvSKV1 in SKV) was completely inserted into the circular chromosome (from 125,709 bp to 393,697 bp) of SPU B805, while the DNA fragment of plasmid 2 (belonging to strain Hbe602, WSH-001 and Y25), was completely lost in SPU B805.

### Genome annotation of *K*. *vulgare* SPU B805

To facilitate the gene function analysis, the genes on chromosome and plasmid 1 of the four published *K*. *vulgare* strains (SKV, Hbe602, WSH-001 and Y25) were selected to compare the distribution of COG classification with SPU B805 (see Supplementary Fig. S5). Consequently, in SPU B805, the number of genes related to amino acid transport and metabolism (E) is similar to that of Hbe602 and Y25 and higher than that in SKV and WSH-001. These functional genes encode many transporters, which absorb nutrients from the environment to compensate for its metabolic defects. Besides, the number of genes related to inorganic ion transport and metabolism (P) and translation, ribosomal structure and biogenesis (J) in SPU B805 is more than those of the reported *K*. *vulgare* strains. Inorganic ions are a kind of important cofactor involved in various physiological reactions in cell. The strong translation ability may be helpful to biosynthesize protein in *K*. *vulgare* SPU B805.

On the basis of the COG and RAST annotation, the genes on plasmid 2 of strain Hbe602, WSH-001 and Y25 account for about 7% of the genome. A majority of them are related to amino acid transport and metabolism (E), and assigned as “an ABC transporter” (Supplementary Fig. S5 and Table S1). Besides, some of the genes for carbohydrate metabolism are annotated as gluconate and ketogluconate metabolism. For example, the enzyme gluconate 2-dehydrogenase (GA2DH) can convert 2-KGA into idonate, followed flowing to 6-phosphogluconate (6PG) and subsequently entering the PPP for energy and biomass production^6^, finally decrease the carbon flow to 2-KGA and lower the 2-KGA production. Additionally, one of the L-sorbosone dehydrogenase (SNDH) genes is also found to be located on plasmid 2. The over-expression of SNDH (encoded by plasmid 2) in *K*. *vulgare* Hbe602 produced an obvious byproduct (not identified its chemical structure in the published paper), so as to decrease the 2-KGA yield^15^. Moreover, the key genes unique to ED pathway, 2-keto-3-deoxy-6-phosphogluconate aldolase (Eda) and phosphogluconate dehydratase (Edd), are annotated to be located on plasmid 2. The deletion of edd and eda genes from *Gluconobacter oxydans* 621H resulted in a lower overall sugar uptake by cells in the cytoplasm, and led to more sugar being left in the periplasm and converted to the end-product (2-keto-gluconate) with high yield^16^. The loss of ED pathway maybe has a similar effect on 2-KGA production in *K*. *vulgare*. Fortunately, these genes (GA2DH, SNDH, Eda and Edd) located on the indigenous plasmid 2, are all absent from the genome of *K*. *vulgare* SPU B805, which may be a reason for its high 2-KGA production. Therefore, presumably, elimination of the above genes or indigenous plasmid 2 is an effective method to enhance 2-KGA production by *K*. *vulgare*. Moreover, to construction an XFP-PTA pathway as described by Wang, *et al*.^17^ to decrease the carbon flux towards ED pathway with lower carbon loss is another pathway to promote the 2-KGA yield.

**Figure 2:**
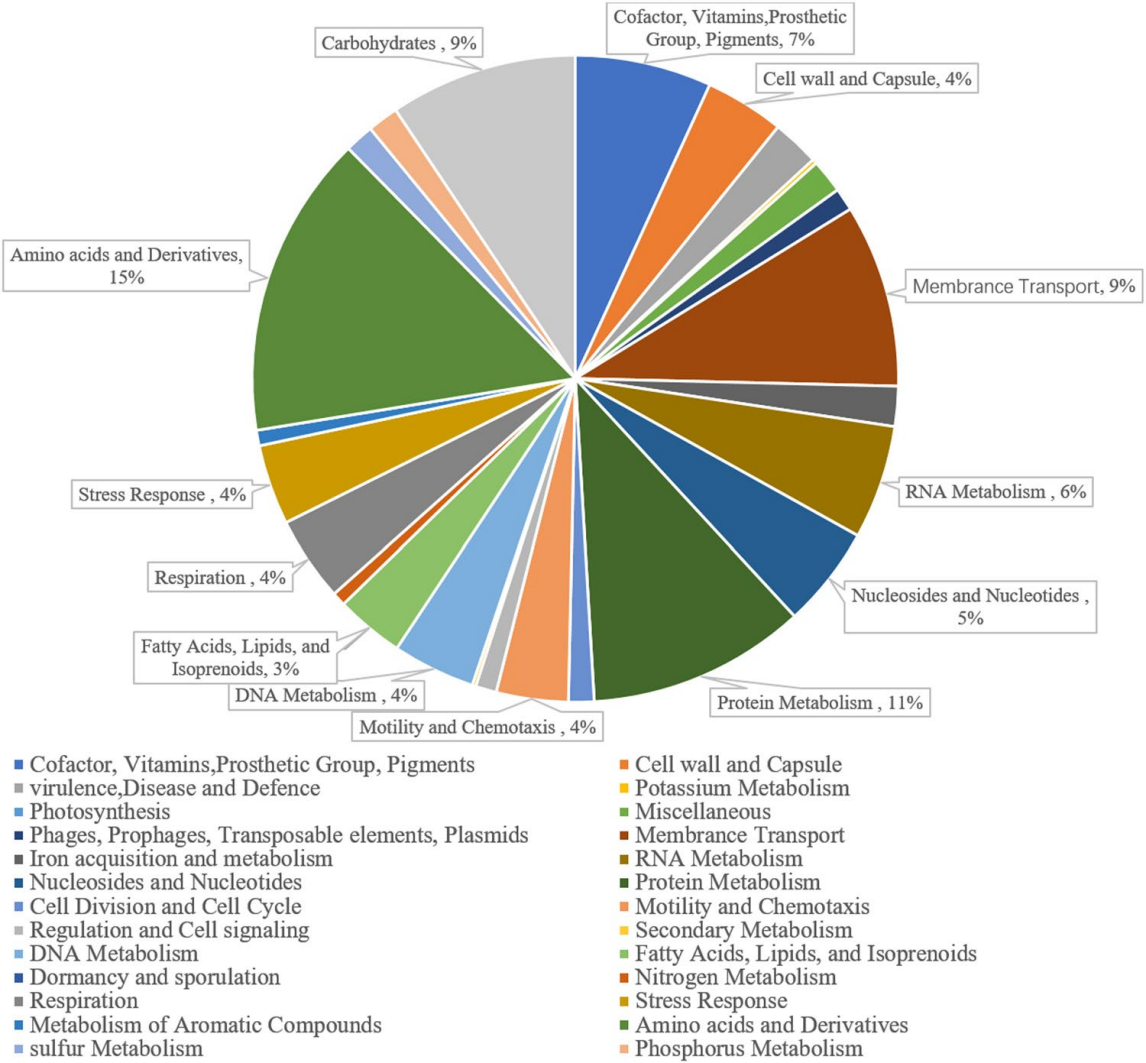
Gene distributions of metabolic subsystems of *K*. *vulgare* SPU B805.

**Figure 3:**
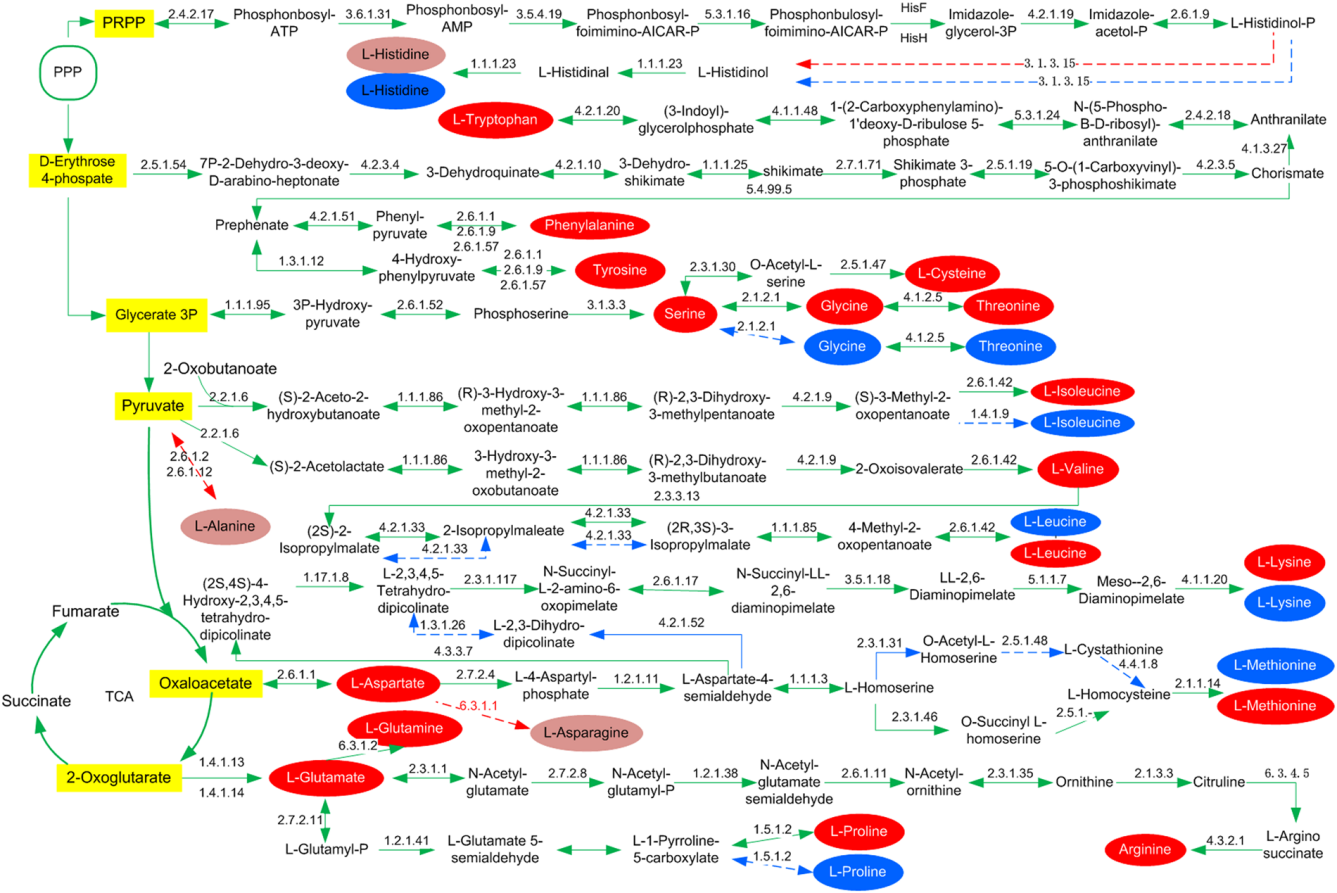
Reconstruction of the amino acid biosynthesis pathway of *K*. *vulgare*. Red ovals and dark red ovals represent the complete and incomplete amino acids biosynthesis pathway of *K*. *vulgare* SPU B805. The blue ovals represent the incomplete biosynthesis pathway of amino acids of *K*. *vulgare* WSH-001. The dotted lines indicate the genes related to the amino acid biosynthesis are absent.

Moreover, a total of 2933 coding sequences were obtained by RAST annotation in strain SPU B805, which belonged to 26 subsystems, including cofactor, membrane transport metabolism, nucleotides metabolism, protein metabolism, regulation and cell signaling, amino acids and carbohydrate metabolism, and so on. Among them, genes regarding to amino acid metabolism were accounted for the highest proportion (15%) (Fig. 2 and Supplementary Table S2). However, the genes coding histidinol-phosphate (EC 3.1.3.15), leucine-alanine transaminase (EC 2.6.1.12), alanine transaminase (EC 2.6.1.2) and asparagine synthetase (EC 6.3.1.1) were found to be absent in strain SPU B805, which resulted in the incomplete biosynthesis pathway of histidine, alanine and asparagine (Fig. 3). While in strain WSH-001, the biosynthesis pathways of more amino acids, such as histidine, glycine, lysine, proline, threonine, methionine, leucine and isoleucine, were deficient due to absence of one or more key enzymes^7^. So the biosynthetic pathways of amino acid in *K*. *vulgare* SPU B805 are more complete than those in the reported *K*. *vulgare* strain WSH-001. Previous study showed that glycine, serine, threonine and proline were important factors for 2-KGA biosynthesis, and threonine affected cell growth significantly^8^. Glycine and serine are donors of one-carbon units in one-carbon metabolism, and threonine can be converted into glycine and acetyl-CoA. The impairment of these amino acids biosynthesis pathways will weaken the biosynthesis of nucleic acid and protein, and the biogenesis methyl group. Moreover, proline can decrease the destruction of microbial cells resulted by the osmotic stress at high 2-KGA concentrations^18^. Therefore, presumably, the more complete biosynthesis pathways in strain SPU B805 may contribute to the growth of *K*. *vulgare* and the accumulation of 2-KGA. Besides, the membrane transport genes were abundant in strain SPU B805, which might help to transport and absorb the nutrients and metabolites released by *B*. *megatherium*^1^.

### Construction of carbohydrate metabolic network

Generally, the prokaryote carbohydrate metabolic pathways are diverse, including the EMP pathway, ED pathway and PPP pathway. However, the genomic annotation revealed that the genes coding 6-phosphofructokinase (EC 2.7.1.11, Pfk), 2-keto-3-deoxy-6-phosphogluconate aldolase (EC 4.1.2.14, Eda) and phosphogluconate dehydratase (EC 4.2.1.12, Edd) were missing in strain SPU B805. Because Pfk is an enzyme specific to the EMP pathway, the absence of *pfk* leads to the incompleteness of the EMP route even though all the other enzymes are present. Similarly, Eda and Edd are the key enzymes unique to the ED pathway, therefore the absence of *eda* and *edd* imply the ED pathway is disabled in strain SPU B805. Thus, the PPP pathway was speculated as the main carbohydrate metabolic pathway in strain SPU B805.

The EMP pathways of strain WSH-001, Y25 and Hbe602 were also analyzed based on their published genome, and found to be incomplete because of the absence of gene *pfk* (Table 3), therefore, we speculated that the ED and PPP pathways were the main carbohydrate metabolic routes. However, for strain SKV, the gene *pfk*, *eda* and *edd* were all absent from the genome, so presumably the carbohydrate metabolism was mainly through the PPP pathway. To sum up, there are two types of carbohydrate metabolic pathways in *K*. *vulgare*. One is simultaneously through the ED and PPP pathways in strain WSH-001, Y25 and Hbe602, the other one is only through the PPP pathway in strain SKV and SPU B805. The loss of the ED pathway had no negative effective on 2-KGA production (Table 1) further confirmed that the ED pathway might be negligible in the cell growth and 2-KGA accumulation, thus the PPP pathway played an important role in the cytoplasmic carbohydrate metabolism in *K*. *vulgare*.

**Table 3:** The major carbohydrate metabolic genes in 2-KGA-producing strains of *K*. *vulgare*.

Enzyme	Pathway	EC number	*K*. *vulgare* SPU B805	*K*. *vulgare* SKV	*K*. *vulgare* Hbe602	*K*. *vulgare* WSH-001	*K*. *vulgare* Y25
Glk	EMP	1.1.5.2	KVC_0743	KvSKV_08250	KVH_08280	KVU_0226	EIO_1719
Pfk	EMP	2.7.1.11	Null	Null	Null	Null	Null
Fda	EMP	4.1.2.13	KVC_1210	KvSKV_05540	KVH_05570	KVU_0671	EIO_1171
TpiA	EMP	5.3.1.1	KVC_1436	KvSKV_06720	KVH_06750	KVU_0894	EIO_1404
Gap	EMP	1.2.1.12	KVC_2250	KvSKV_10850	KVH_10930	KVU_1674	EIO_2115
Pgk	EMP	2.7.2.3	KVC_0927	KvSKV_05545	KVH_05575	KVU_0672	EIO_1172
Pgm	EMP	5.4.1.12	KVC_2728	KvSKV_13275	KVH_13365	KVU_2126	EIO_2619
PykA	EMP	2.7.1.40	KVC_0447	KvSKV_01710	KVH_01705	KVU_2550	EIO_0203
Fbp	EMP	3.1.3.11	KVC_2446	KvSKV_12525	KVH_12605	KVU_1985	EIO_2462
Edd	ED	4.2.1.12	Null	Null	KVH_edd*	KVU_PB0156	EIO_3349
Eda	ED	4.1.2.14	Null	Null	KVH_eda*	KVU_PB0157	EIO_3350
Zwf	PPP	1.1.1.49	KVC_1674	KvSKV_07920	KVH_07950	KVU_1124	EIO_1652
Pgl	PPP	3.1.1.31	KVC_1673	KvSKV_07915	KVH_07945	KVU_1123	EIO_1651
Gnd	PPP	1.1.1.44	KVC_0890	KvSKV_03940	KVH_03970	KVU_0365	EIO_0834
RpiA	PPP	5.3.1.6	KVC_0880	KvSKV_03890	KVH_03920	KVU_0356	EIO_0823
Rpe	PPP	5.1.3.1	KVC_2798	KvSKV_03940	KVH_13715	KVU_2194	EIO_2692
TktA	PPP	2.2.1.1	KVC_2247	KvSKV_10835	KVH_10920	KVU_1671	EIO_2112
Tal	PPP	2.2.1.2	KVC_1789	KvSKV_RS08555	KVH_08530	KVU_1236	EIO_1771
AceE	TCA	1.2.4.1	KVC_1207, 1208	KvSKV_05525	KVH_05560	KVU_0668, 0669	EIO_1168, 1169
GltA	TCA	2.3.3.1	KVC_2235	KvSKV_10775	KVH_10860	KVU_1659	EIO_2099
Acn	TCA	4.2.1.3	KVC_0695, 1409	KvSKV_06590	KVH_06620	KVU_0869	EIO_1377
Icd	TCA	1.1.1.42	KVC_1885	KvSKV_08965	KVH_09010	KVU_1326	EIO_1866
SucA	TCA	1.2.4.2	KVC_0103	KvSKV_01120	KVH_01115	KVU_2455	EIO_0115
SucDC	TCA	6.2.1.5	KVC_0104	KvSKV_11895	KVH_01125	KVU_2456, 2457	EIO_0116, 0117
SdhA	TCA	1.3.5.4	KVC_0109-0111, 0115	KvSKV_01155	KVH_01150	KVU_2460-2462	EIO_0120-0122, 0126
FumC	TCA	4.2.1.2	KVC_1668	KvSKV_07885	KVH_07915	KVU_1117	EIO_1646
Mdh	TCA	1.1.1.37	KVC_0106	KvSKV_01135	KVH_01130	KVU_2458	EIO_0118

Abbreviations: Glucokinase (Glk), 6-Phosphofructokinase (Pfk), Fructose-1,6-bisphosphate aldolase (Fda), Triosephosphate isomerase (TpiA), Glyceraldehyde-3-phosphate dehydrogenase (Gap), Phosphoglycerate kinase (Pgk), Phosphoglycerate mutase (Pgm), Pyruvate kinase (PykA), Fructose-1,6-bisphosphatase (Fbp), 6-phosphogluconate dehydratase (Edd), 2-keto-3-deoxy-6-phosphogluconate aldolase (Eda), Glucose-6-phosphate 1-dehydrogenase (Zwf), 6-phosphogluconolactonase (Pgl), 6-phosphogluconate dehydrogenase (Gnd), Ribose-5-phosphate isomerase (RpiA), Ribulose-5-phosphate epimerase (Rpe), Transketolase (TktA), Transaldolase B (Tal), Pyruvate dehydrogenase (aceE), Citrate synthase (GltA), Aconitate hydratase (Acn), Isocitrate dehydrogenase (Icd), 2-oxoglutarate dehydrogenase (SucA), Succinyl-CoA synthetase (SucDC), Succinate dehydrogenase (SdhA), Fumarate hydratase (FumC), Malate dehydrogenase (Mdh).

* indicated that the gene sequence in Hbe602 was 100% identity with the gene encoding Edd and Eda in WSH-001, but the two genes were not annotated by the original authors, so the two genes were annotated as KVH_edd, and KVH_eda in this study.

### Carbohydrate metabolism analysis based on transcriptome

**Figure 4:**
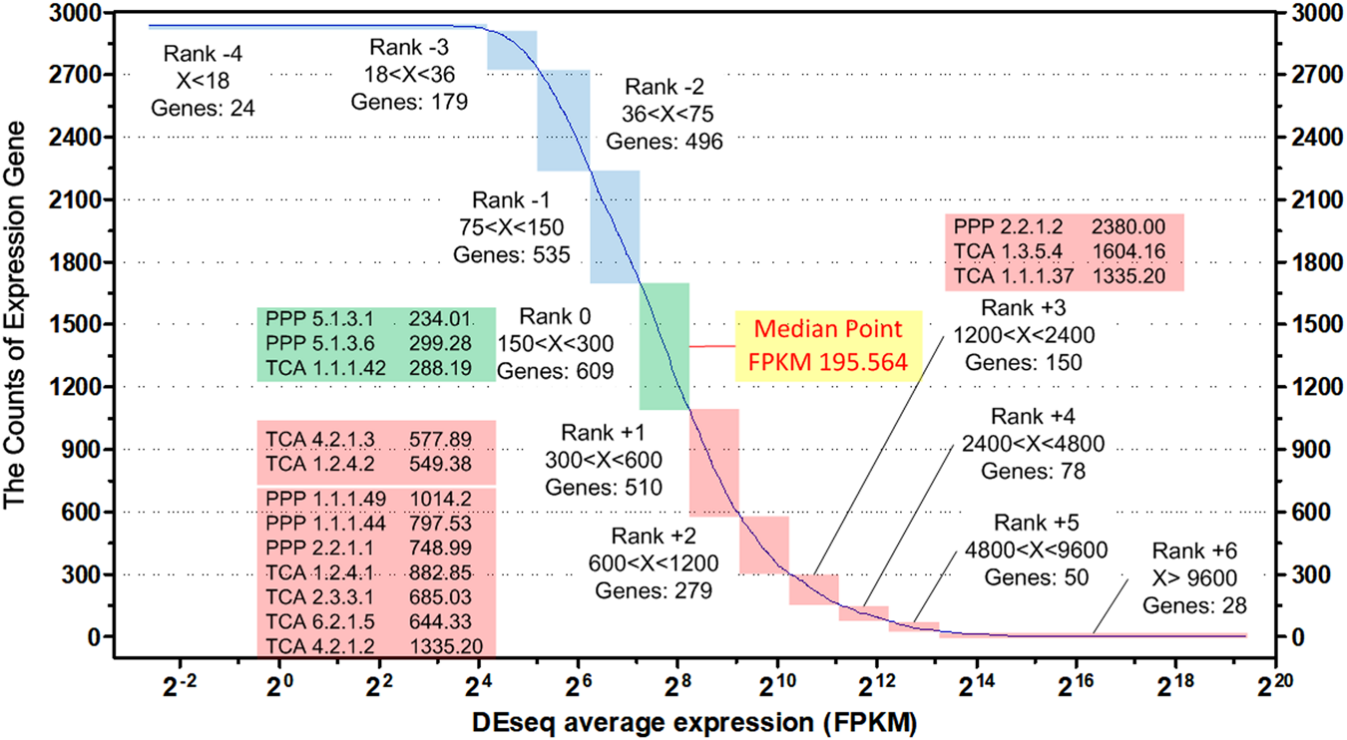
Transcriptome information of *K*. *vulgare* SPU B805. X axial represents the FPKM of *K*. *vulgare* SPU B805, Y axial represents the counts of expressed genes. The blue and pink panels indicate the FPKM is below and above the median point, respectively. The FPKM between 150 and 300, 300 and 600, 600 and 1200, 600 and 1200, 1200 and 2400, 2400 and 4800, 4800 and 9600 was defined as Rank 0, +1, +2, +3, +4, +5. The FPKM between 75 and 150, 36 and 75, 18 and 36 was defined as Rank −1, −2, and −3. The FPKM above 9600 and below 18 was defined as Rank +6 and Rank −4.

The abundance of FPKM (fragments per kilo bases of million fragments) was employed to represent the gene transcriptional level^19^. As for strain SPU B805, the whole FPKM was divided into 11 grades (from Rank −4 to Rank +6), and the median point was 195.564 (Fig. 4). The FPKM of the key genes related to PPP pathway and TCA cycle (Zwf, EC 1.1.1.49; Gnd, EC 1.1.1.44; Rpe, EC 5.1.3.1; RpiA, EC 5.3.1.6; TktA, EC 2.2.1.1; Tal, EC 2.2.1.2; GltA, EC 2.3.3.1; AceE, EC 1.2.4.1; Acn, EC 4.2.1.3; Icd, EC 1.1.1.42; SucA, EC 1.2.4.2; SucDC, EC 6.2.1.5; SdhA, EC 1.3.5.4; FumC, EC 4.2.1.2; Mdh, EC 1.1.1.37) were marked out in Fig. 4, and it was noteworthy that all of the genes related to carbohydrate metabolism (PPP, TCA) were transcribed at higher levels than the median point. However, no transcription signals of *pfk*, *ga2dh*, *edd* and *eda* could be detected, which confirmed that these genes did not exist in the genome of strain SPU B805, and indicated that the EMP and ED pathway were disabled. Because the PPP pathway, ED pathway and EMP pathway were three parallel carbon metabolic routes before TCA cycle, therefore, the only existing complete PPP pathway was assigned as the major and important cytoplasmic carbohydrate decomposition pathway linked with TCA cycle in strain SPU B805.

**Figure 5:**
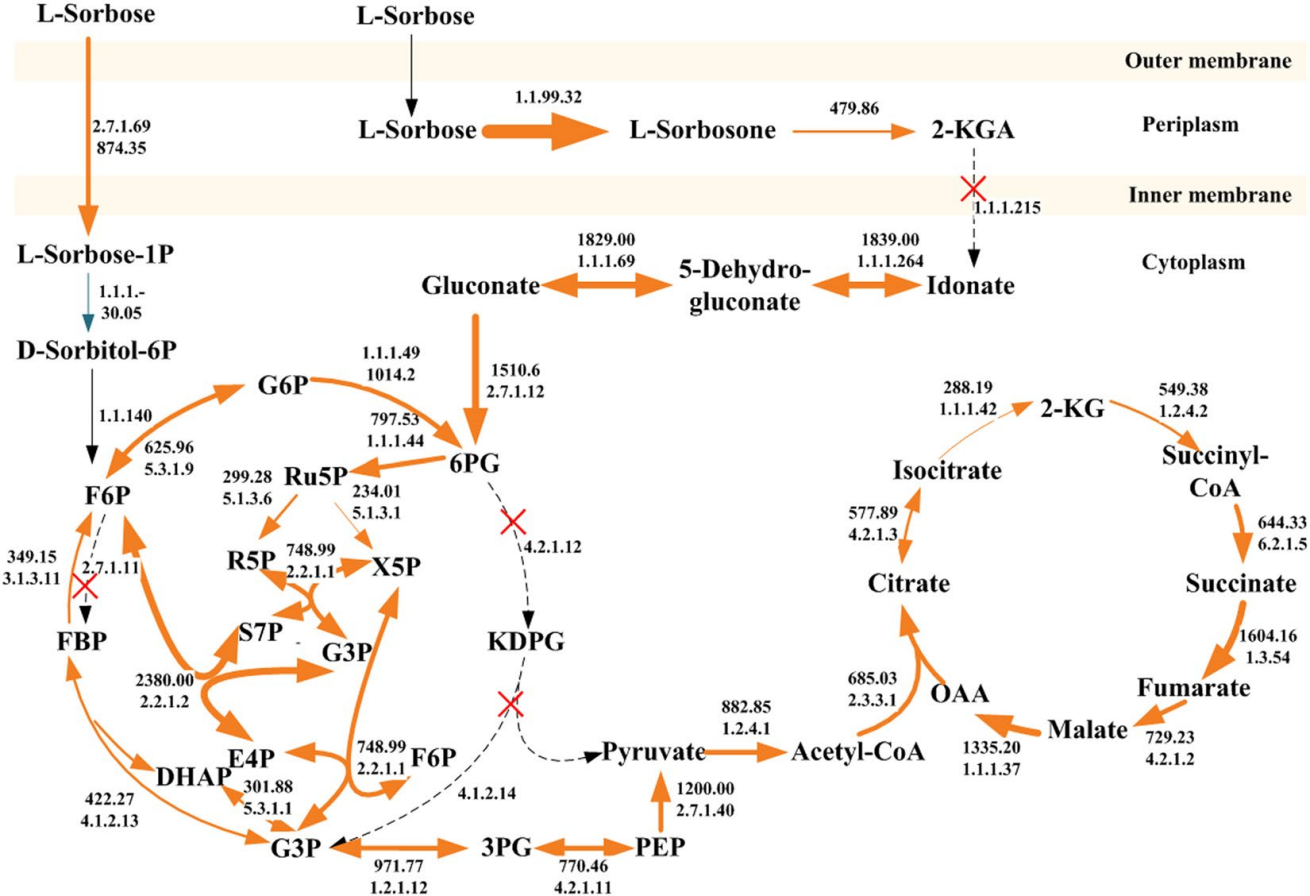
Overview of the average FPKM of carbohydrate metabolism related genes in *K*. *vulgare* SPU B805. Blue lines and orange lines indicate the average FPKM is lower and higher than the median point, respectively. Dotted lines indicate no transcription signal is detected. The thickness of lines represents the FPKM level. The figures above or below lines represent the EC number of the enzyme and the average FPKM.

In fermentation, the FPKM of the five *sdh* (KVC_2764, KVC_2744, KVC_0718, KVC_1927, KVC_0337) (the average FPKM value 24691.32, 10501.89, 2078.73, 750.78, 220.99) were obviously higher than those of the central carbon degradation related genes, which partially explained that the majority of L-sorbose was converted to 2-KGA in strain SPU B805. Although the gene *sndh* (KVC_0605, the average FPKM value 479.86) had a relative lower transcriptional level, it did not affect the 2-KGA production rate because SDH was a dual-function enzyme, which could oxidize L-sorbose to L-sorbosone and further oxidize to 2-KGA^20^. Therefore, the central carbon metabolism, as well as the transcription level (the average FPKM value), were put together in Fig. 5. Strain SPU B805 possesses two separated modes for L-sorbose catabolism. A major part of L-sorbose is oxidized to L-sorbosone in the periplasm by the membrane-bound SDH, and further to 2-KGA by SDH and SNDH. The produced 2-KGA can’t be further decomposed to idonate because of the absence of plasmid 2 (so absence of gene *ga2dh*), therefore the 2-KGA production can reach a higher level in SPU B805 than that in Hbe602 and WSH-001. A minor part of L-sorbose as carbon source is catabolized to F6P and subsequently to 6PG in cytoplasm, and then enters the PPP pathway for energy and biomass production. In the oxidative branch, 6PG is converted into ribulose 5-phosphate (Ru5P), carbon dioxide and NADPH. NADPH is the major reducing power used to maintain the redox balance under stress situation^21^. The formed Ru5P is converted to R5P by ribose 5-phosphate isomerase (RpiA, EC 5,3,1,6) or X5P by ribulose 5-phosphate epimerase (Rpe, EC 5.1.3.1) in the non-oxidative branch. R5P and X5P undergo carbon rearrangement to generate sedoheptulose 7-phosphate (S7P), glyceraldehyde 3-phosphate (G3P) and erythorse 4-phosphate (E4P). R5P and E4P, as the precursors, were used for the biosynthesis of RNA, DNA, as well as aromatic amino acids^13^.

### RT-qPCR validation of the genes related to carbohydrate metabolism

The key genes in PPP pathway, *zwf* (KVC_1674), *gnd* (KVC_0890), transketolase (KVC_2247; *tkt*A) and transaldolase B (KVC_1789; *tal*) were conducted by RT-qPCR. Compared with internal standard (*pol*A), all of the four genes showed a relative high transcription level, and the changed trends in different time-points were in accordance with the transcriptome results (see Supplementary Fig. S6). The gene transcription level assayed by RNA-seq and RT-qPCR showed high correlation, indicating the reliability of the RNA-seq analysis.

## Discussion

For a long time, researchers have mainly concentrated on elucidating the symbiosis mechanism of *K*. *vulgare* and *B*. *megatherium*, however, few studies have been explored on the underlying mechanism for the differences in central carbon metabolic pathways and 2-KGA yield of *K*. *vulgare*. In this study, the genome of *K*. *vulgare* SPU B805 was completely sequenced and the metabolic network was reconstructed, which provided a new insight into the carbohydrate metabolic specificity. *K*. *vulgare* SPU B805 was the first 2-KGA-producing strain without any plasmid. Its genome was similar to *K*. *vulgare* SKV except for some insertion, dislocation and rearrangement. The genome annotation showed that except for histidine, alanine and asparagine, the biosynthetic pathway of other amino acids was complete in strain SPU B805, which had significantly integral function than that in WSH-001 (eight amino acid biosynthesis pathways incomplete)^6^. Liu, *et al*.^7^ reported that glycine, proline, threonine and isoleucine play vital roles in *K*. *vulgare* growth and 2-KGA production, and the addition of these amino acids increased the 2-KGA productivity by 20.4%, 17.2%, 17.2% and 11.8%, respectively. Zhang, *et al*.^8^ also determined the key factors affected 2-KGA fermentation by orthogonal design experiments, and the results indicated that glycine and threonine were the key components affecting the mixed cell growth; serine, glycine, and proline were the key components that affected the 2-KGA production. Therefore, the more complete amino acid biosynthetic pathway, the more contribution to the high 2-KGA production of *K*. *vulgare* SPU B805. Moreover, the absence of the SNDH on plasmid 2 can avoid the unknown byproduct in the formation of 2-KGA. Besides, the gene *ga2dh*, responsible for the subsequent decomposition of 2-KGA to idonate, was also absent from strain SPU B805, which was a very advantageous factor for the 2-KGA accumulation. Additionally, the higher transcriptional level of five *sdh* (KVC_2764, KVC_2744, KVC_0718, KVC_1927, KVC_0337) helps to explain the high L-sorbose conversion ability of strain SPU B805.

The genome and transcriptome analysis showed that the central carbon metabolism of *K*. *vulgare* was versatile. Strain WSH-001, Y25 and Hbe602 contained all the genes involved in the ED, the PPP and the TCA cycle, meaning that these pathways were complete^2–4^. While the *pfk* gene was absent from strain WSH-001, Y25 and Hbe602 genome, indicating that the EMP pathway was nonfunctional in these strains. Whereas, in strain SPU B805 and SKV, all the genes referred to the PPP and the TCA cycle were found, meaning that the PPP and the TCA pathway were complete^5^. The *pfk*, as well as *eda* and *edd* genes were simultaneously missing from the genome of strain SPU B805 and SKV, which suggested that neither of the EMP and the ED pathway were functional. Therefore, the PPP pathway is the exclusively pathway for cytoplasmic carbohydrate decomposition in strain SPU B805 and SKV. In strain WSH-001, Y25 and Hbe602, the ED and the PPP pathways are paralleled for cytoplasmic carbohydrate catabolism. Although the ED pathway was complete in strain WSH-001, Y25 and Hbe602 genome, the carbon flux through this pathway is presumably negligible because of the lower ATP productivity ability of the ED pathway^10^. Moreover, in *G*. *oxydans* 621H, the deletion of *gnd* to inactive the PPP pathway resulted in a reduced final biomass and a decreased end-product 2-keto-gluconate, whereas, the inactivation of the ED pathway by deleting the *edd*-*eda* genes caused a lower overall sugar uptake by cells for the formation of biomass and energy in the cytoplasm, and led to a larger fraction of the sugar being converted to the final product of 2-keto-gluconate in the periplasm^16^. This research indicated that the PPP pathway was of the major importance for cytoplasmic carbohydrate catabolism, whereas the ED pathway is dispensable. The loss of ED pathway maybe has a similar effect on 2-KGA production in *K*. *vulgare*. Although higher ATP productivity in the EMP pathway than in the ED pathway, the EMP requires more enzymes to sustain the equivalent carbon flux^10^. If the protein synthesis is a limiting factor for cell growth, the bacterium usually does not synthesize additional protein only for one more energy^22,23^.

The PPP pathway, as a fundamental component in cellular metabolism, plays an important role in maintain carbon homoeostasis^13^. The PPP pathway can provide various intermediate metabolites for the biosynthesis of nucleotide and amino acids, such as G3P, E4P, R5P, S7P, and generate NADPH for the maintenance of redox balance under the stress situations^21^. *K*. *vulgare* as an aerobic bacterium, its growth and metabolism are inevitably subject to reactive oxygen species (ROS)^24,25^, therefore a reducing environment is an essential requirement for cell metabolism. It is well known that NADPH is the major reducing power in cell, and plays an important role in the GSH and thioredoxin metabolism^26–28^. Besides, a large number of anti-oxidant enzymes are coupled with NADPH as cofactor^29^, thus the PPP pathway may help *K*. *vulgare* SPU B805 to defeat intracellular ROS. Moreover, the biomass of *K*. *vulgare* was composed of protein, DNA, RNA, lipids, peptidoglycans, lipopolysaccharides, glycogen and soluble pool^6,30^. It is conceivable that the PPP pathway may provide a variety of intermediates as precursors to satisfy the demands for the biosynthesis of cellular components and facilitate to curb different types of environmental hardship in the process of growth.

## Materials and Methods

### Bacterial strains

Two strains, *K*. *vulgare* SPU B805 (2-KGA-producing strain) and *B*. *megatherium* SPU B806 (the helper strain), which were stored in Microbial Resource Center of Shenyang Pharmaceutical University, were used for 2-KGA fermentation in this study.

### Culture medium and fermentation

The plate medium (P medium) contained 20 g/L L-sorbose, 1 g/L KH_2_PO_4_, 3 g/L yeast powder, 0.2 g/L MgSO_4_, 3 g/L beef extract, 1 g/L urea, 10 g/L peptone, 3 g/L corn steep liquor and 20 g/L agar, pH 6.8. The plates were cultured at 28–30 °C for 4 days. The seed medium (S medium) contained 20 g/L L-sorbose, 1 g/L KH_2_PO_4_, 3 g/L yeast powder, 0.2 g/L MgSO_4_, 12 g/L urea, 10 g/L corn steep liquor, pH 6.8. Seeds were cultured at 28–30 °C, 250 rpm for 18 h. The fermentation medium (F medium) contained 80 g/L L-sorbose, 20 g/L corn steep liquor, 1 g/L KH_2_PO_4_, 0.2 g/L MgSO_4_, 12 g/L urea. The fermentation was conducted in 5-L fermenter containing 3 L F medium and 300 mL co-inoculated seeds of *K*. *vulgare* SPU B805 and *B*. *megatherium* SPU B806. The fermentation pH was maintained automatically at 6.8–7.0 by addition of 30% (m/v) Na_2_CO_3_ solution. The temperature was controlled at 30 °C, and the agitation speed was 400 rpm with the air flow rate of 1.5 L/min.

To calculate the Colony Forming Units (CFUs) of *K*. *vulgare*, the fermentation broth was diluted and spread on P plate, and the growth curve was drawn according to the CFU/mL. Meanwhile, the cell density was measured spectrophotometrically at 660 nm.

### Genome sequencing and assembly

*K*. *vulgare* SPU B805 was cultured in 250 mL flasks with 50 mL S medium at 30 °C for 18 h at 220 rpm. The genome of strain SPU B805 was extracted with the TIANamp Genomic DNA Kit (Tiangen Biotech (Beijing) Co., Ltd.) according to the instruction. The concentration and purity of DNA sample were qualified by Agilent 2100 BioAnalyzer (Agilent Technologies, USA). The integrity of genomic DNA was detected by agarose gel electrophoresis. The DNA samples with a 260/280 nm absorbance ration of 1.8–2.0 and a 260/230 nm absorbance ration of 2.0–2.2 were considered pure and then used for the library construction and sequencing.

The complete genome sequence was determined by the Chinese National Human Genome Center (Shanghai, China) using 454 single-end sequencing technology. It yielded a total of 116,450 reads with a 17.29-fold coverage of the genome. The reads were assembled into 26 large contigs (>500 nucleotides) and 3 small contigs (100–500 nucleotides) by using the 454 Newbler assembler (454 Life Sciences, Branford, CT). The relationships between the contigs were determined by ContigScape^31^, and the gaps between the contigs were closed by PCR amplification, followed by DNA sequencing. Finally, the contigs were assembled into a circular chromosome, and no plasmid was found. The complete genome of SPU B805 had an error rate of less than 0.2 in a 10-kb sequence as determined by sequence assembly and quality assessment using the Phred^32^/Phrap^33^/Consed^34^ software package.

### Genome annotation

Protein-coding genes were predicted by combining the results of Glimmer 3.02^35^ and ZCURVE^36^, followed by manual inspection. Each gene was functionally classified by assigning a Cluster of Orthologous groups (COG) number. Both tRNA and rRNA genes were identified by tRNA scan-SE^37^ and RNAmmer^38^, respectively. Functional annotations of ORFs were performed by searching against public databases, including NCBI (http://www.ncbi.nlm.nih.gov/), KEGG^39^ and SWISS-Prot^40^. Basic metabolic pathways were reconstructed by KEGG and MetaCyc^41^. The Rapid Annotation using Subsystem Technology (RAST) server^42^ was used to re-annotate the genome of *K*. *vulgare* SPU B805. Then, RAST annotation was put through the Model SEED pipeline to obtain the initial network reconstruction^43^.

### Transcriptome sequencing

*K*. *vulgare* SPU B805 and *B*. *megatherium* SPU B806 were co-cultured in 5-L fermenter. The fermentation processes of 0 h (seed), 8 h, 16 h, 24 h, 32 h and 40 h were chosen to represent different growth phase. The co-culture samples were subjected to a centrifugation of 4,000 rpm for 5 min at 4 °C to eliminate the cells of *B*. *megatherium* SPU B806. The RNA of *K*. *vulgare* SPU B805 was extracted with the RNAprep Pure Bacteria Kit (Tiangen, Beijing, China). The concentration and purity of RNA sample were qualified by a Agilent 2100 BioAnalyzer (Agilent Technologies, USA). The integrity was monitored on 1% agarose gels. The RNA samples with a 260/280 nm absorbance ration of 1.8–2.1 were considered pure, and a rRNA ration (28S/18S) ≥1.5 and RIN ≥7 were considered integrity. The NEBNext Ultra RNA Library Prep Kit (NEB, USA) for Illumina was used to construct sequencing libraries. The rRNA was removed from total RNA using a Ribo-Zero Magnetia Kit (Epicentre, USA) to enrich the mRNA and then the cDNA was synthesized. The library preparation was sequenced on Illumina Hiseq 2500 platform. The genome of *K*. *vulgare* SPU B805 was used as a reference.

### Analyses of 2-KGA and L-sorbose

The concentration of 2-KGA and L-sorbose in the fermentation broth were analyzed by the High-Performance Liquid Chromatography (HPLC) (Waters Corp., Massachusetts, USA) using an amino column (Agilent Zorbax NH2) with an ultraviolet detector at 210 nm. Cetonitrile-KH_2_PO_4_ (5%/95%, v/v) was used as mobile phase with a flow rate of 0.6 mL/min.

### Real time quantitative PCR

The primers used for real time quantitative PCR (RT-qPCR) were listed in Supplementary Table S3. RT-qPCR was conducted with the GoTaq qPCR Master Mix (Promega (Beijing) Biotech Co., Ltd) on a Stratagene Mx3000P (Agilent Technologies Inc, USA). The DNA polymerase I (KVC_2082, *pol*A) gene was used as an internal standard. The fold change of gene expression was calculated by the 2^−ΔΔCT^ method^44^.

## Electronic supplementary material

Supplementary material
