# Supplementary material for: Reconstruction and analysis of carbon metabolic pathway of Ketogulonicigenium vulgare SPU B805 by genome and transcriptome
Source: Sci Rep. 2018 Dec 13;8:17838. doi: 10.1038/s41598-018-36038-3 (PMC6293013; doi:10.1038/s41598-018-36038-3)
Supplement: Supplementary file 1 — Supplementary material [file 41598_2018_36038_MOESM1_ESM.docx]

**Supplementary material**

**Reconstruction and analysis of carbon metabolic pathway of *Ketogulonicigenium vulgare* SPU B805 by genome and transcriptome**

Cai-Yun Wang ^a^, Ye Li ^a, b^, Zi-Wei Gao ^c^, Li-Cheng Liu ^a^, Ying-Cai Wu ^a^, Meng-Yue Zhang ^a^, Tian-Yuan Zhang ^a^, Yi-Xuan Zhang ^a,^ *

^a^ School of Life Science and Biopharmaceutics, Shenyang Pharmaceutical University, Shenyang, 110016, China.

^b^ Northeast Pharmaceutical Group Co., Ltd, Shenyang, 110026, People's Republic of China

^c^ Department of Biotechnology, School of Engineering, Nagoya University, Furo-cho, Chikusa-ku, Nagoya 464-8603, Japan

* Corresponding Author at: School of Life Science and Biopharmaceutics, Shenyang Pharmaceutical University, 103 Wenhua Road, Shenyang, Liaoning, 110016, China. Tel: +86-024-23986576. Fax: +86-024-23986576. E-mail: [zhangyxzsh@163.com](mailto:zhangyxzsh@163.com)


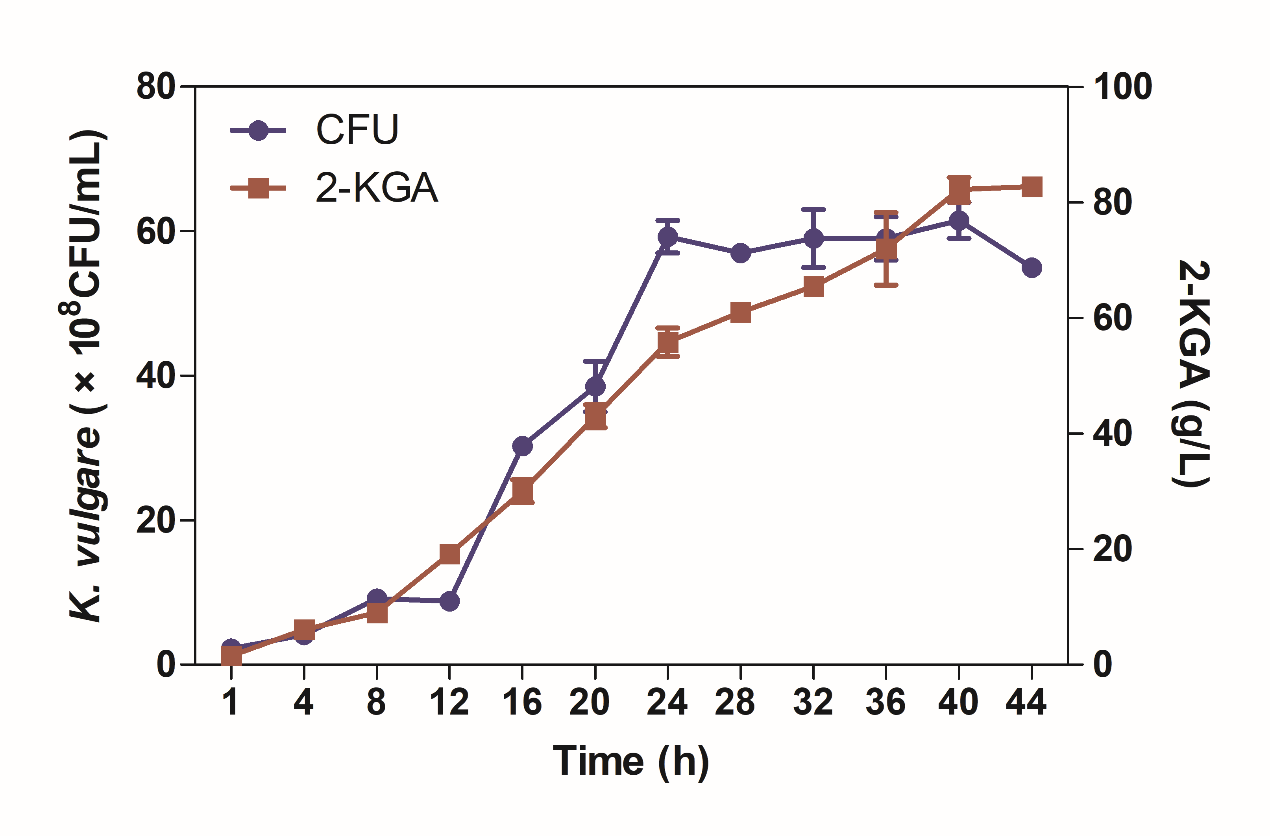


**Figure S1 Fed-batch fermentation of *K. vulgare* SPU B805.** Error bars represents the standard deviation of three biological replicates.


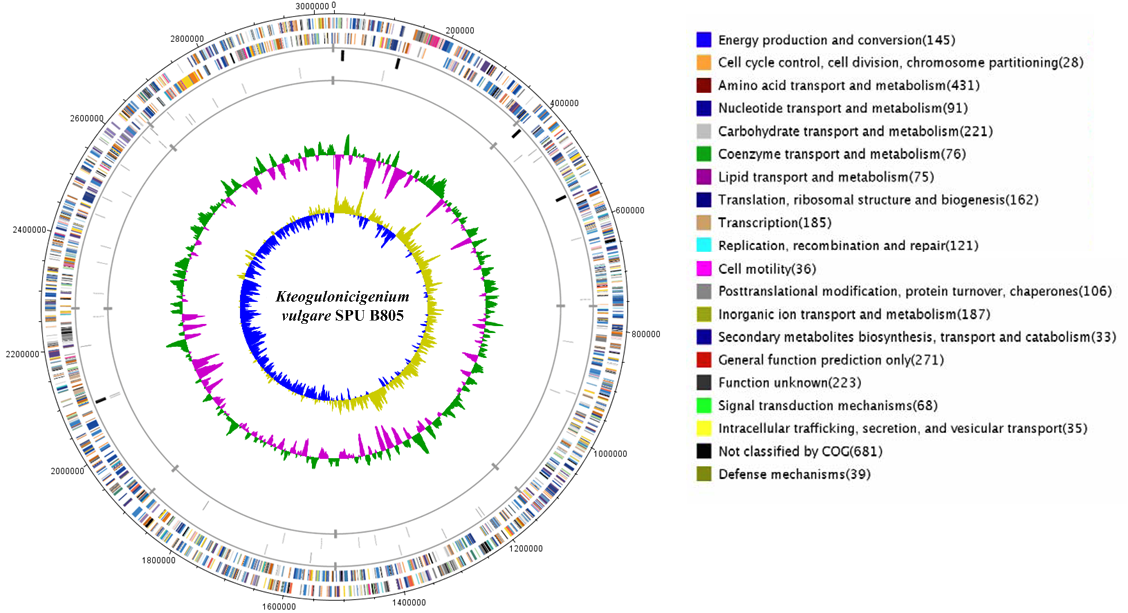


**Figure S2 The genome schematic of *K. vulgare* SPU B805.** From outside to inside: Circle 1 represents the COG annotated gene distribution of the positive strand, which is distinguished by different colors. Circle 2 represents the COG annotated gene distribution of the negative strand. The third circle represents the ncRNA distribution of the positive strand, including tRNA, rRNA, and sRNA. The fourth circle represents the ncRNA distribution of the negative strand. The fifth cycle indicates the GC content, in which the average GC is used as the baseline, outward represents higher than the average and inward represents below the average. The sixth cycle is the GC skew value, in which blue means less than 0, and yellow means greater than 0.


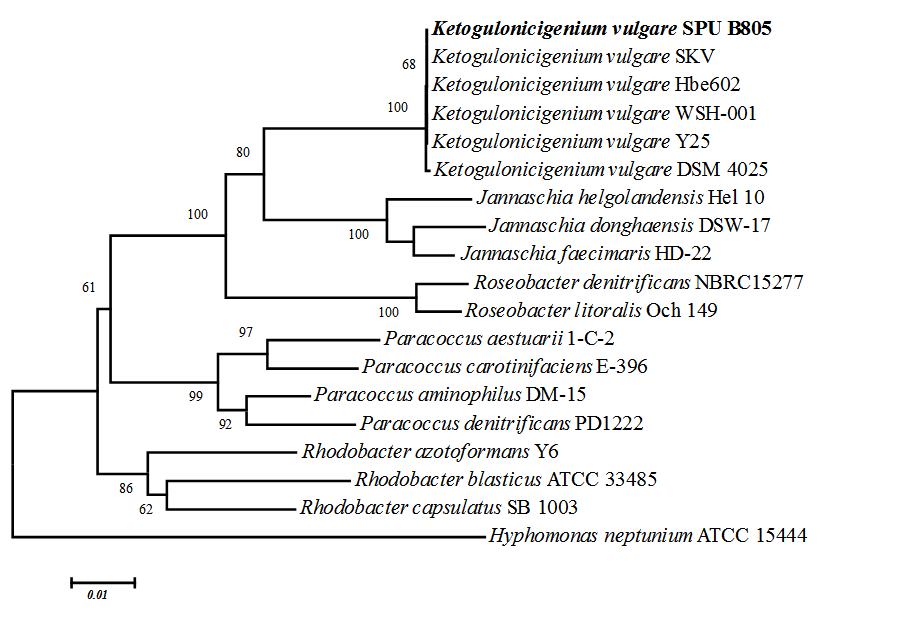


**Figure S3 Phylogenetic analysis of *K. vulgare* SPU B805 with other species.** The neighbor-joining tree was constructed using MEGA5 program. *Hyphomonas neptunium* ATCC 15444 was included as an outgroup.


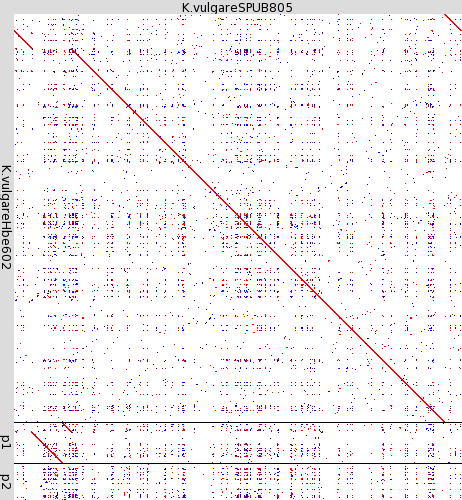

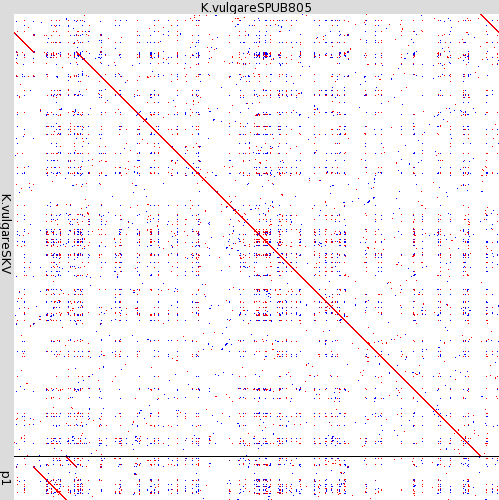
a b


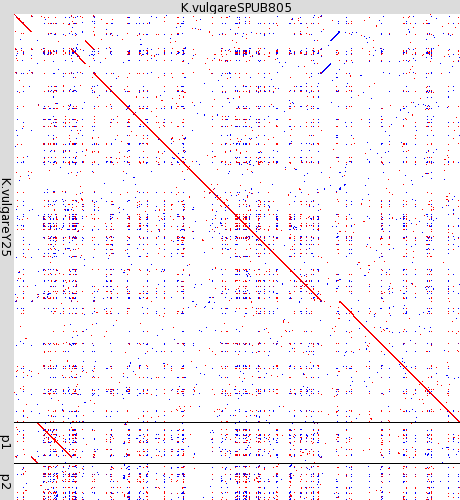

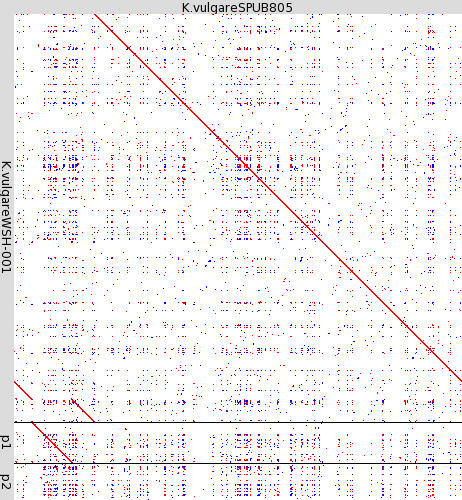
c d

**Figure S4 Genome similarity comparison between *K. vulgare* SPU B805 with the four reported *K. vulgare* strain.** (a), (b), (c) and (d) represents the genome comparison of *K. vulgare* SPU B805 with *K. vulgare* SKV, Hbe602, WSH-001 and Y25, respectively.


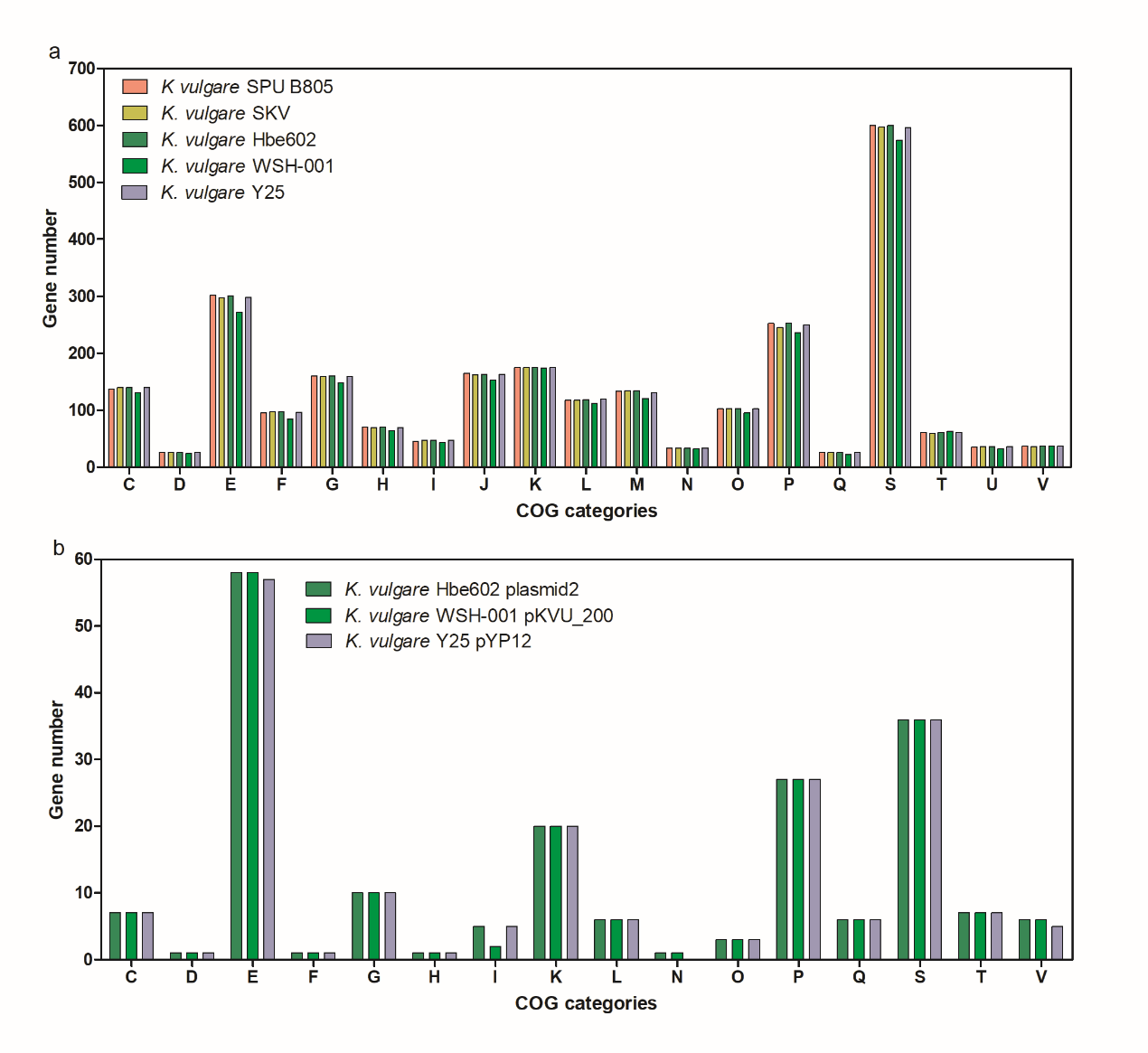


**Figure S5 COG analysis of *K. vulgare* SPU B805 with other strains of *K. vulgare*.** (a) represents the COG annotation of chromosome and plasmid 1 of the four published *K. vulgare* strains (SKV, Hbe602, WSH-001 and Y25) and SPU B805. (b) represents the COG annotation of plasmid 2 for *K. vulgare* Hbe602, WSH-001 and Y25. Abbreviation and function description are shown as follows: J, Translation, ribosomal structure and biogenesis; K, Transcription; L, Replication, recombination and repair; D, Cell cycle control, cell division, chromosome partitioning; V, Defense mechanisms; T, Signal transduction mechanisms; M, Cell wall/membrane/envelope biogenesis; N, Cell motility; U, Intracellular trafficking, secretion, and vesicular transport; O, Posttranslational modification, protein turnover, chaperones; C, Energy production and conversion; G, Carbohydrate transport and metabolism; E, Amino acid transport and metabolism; F, Nucleotide transport and metabolism; H, Coenzyme transport and metabolism; I, Lipid transport and metabolism; P, Inorganic ion transport and metabolism; Q, Secondary metabolites biosynthesis, transport and catabolism; S, Function unknown.


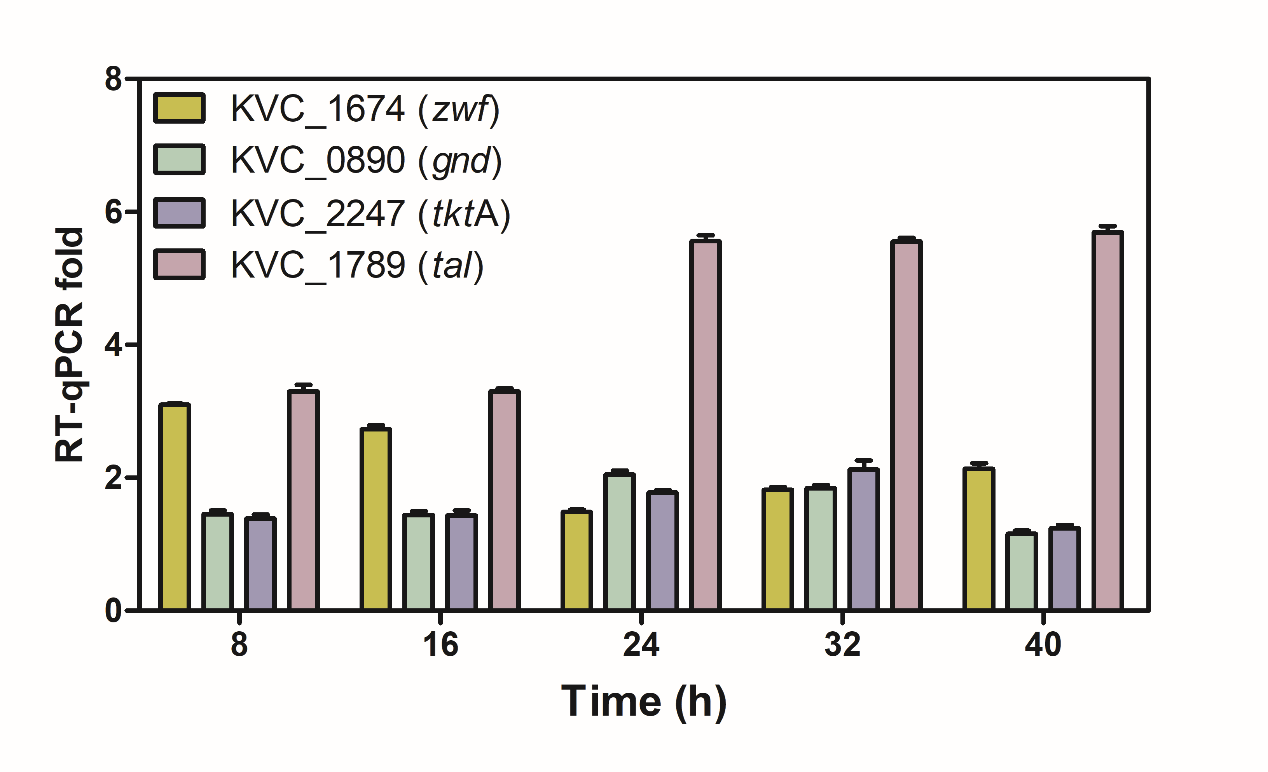


**Figure S6 RT-qPCR analysis of gene expression ratio between samples and internal standard.** Error bars represents the standard deviation of two biological replicates.

**Table S1** **The number of metabolic subsystem related genes in plasmid 2 of *K. vulgare***

| Features | *K. vulgare* Hbe602  plasmind 2 | *K. vulgare* WSH-001 plasmind 2 | *K. vulgare* Y25 plasmind 2 |
| --- | --- | --- | --- |
| Cell wall and capsule | 1 | 1 | 1 |
| Virulence, Disease and Defense | 2 | 1 | 1 |
| ABC Transporters | 39 | 38 | 38 |
| Nucleosides and Nucleotides | 15 | 15 | 18 |
| Protein Metabolism | 2 | 2 | 2 |
| DNA metabolism | 6 | 6 | 6 |
| Dormancy and Sporulation | 2 | 2 | 2 |
| Respiration | 2 | 2 | 2 |
| Stress Response | 3 | 3 | 3 |
| Metabolism of Aromatic Compounds | 17 | 17 | 20 |
| Amino Acids and Derivatives | 13 | 13 | 16 |
| Carbohydrates | 4 | 4 | 4 |

**Table S2 The number of metabolic subsystem related genes in 2-KGA-producing strains of *K. vulgare***

| Features | SPU 805 | SKV | Hbe602 | WSH-001 | Y25 |
| --- | --- | --- | --- | --- | --- |
| Cofactor, Vitamins, Prosthetic groups, Pigments | 150 | 152 | 151 | 151 | 165 |
| Cell wall and Capsule | 87 | 88 | 85 | 87 | 99 |
| virulence, Disease and Defense | 52 | 56 | 53 | 54 | 57 |
| Potassium Metabolism | 5 | 5 | 5 | 5 | 6 |
| Miscellaneous | 37 | 36 | 37 | 36 | 38 |
| Phages, Prophages, Transposable elements, Plasmids | 25 | 25 | 25 | 26 | 26 |
| Membrane Transport | 201 | 201 | 241 | 242 | 247 |
| Iron acquisition and metabolism | 44 | 44 | 44 | 42 | 47 |
| RNA metabolism | 125 | 127 | 125 | 125 | 130 |
| Nucleosides and Nucleotides | 111 | 112 | 126 | 126 | 131 |
| Protein Metabolism | 240 | 242 | 244 | 243 | 228 |
| Cell Division and Cell Cycle | 28 | 32 | 28 | 28 | 31 |
| Motility and Chemotaxis | 79 | 78 | 84 | 88 | 92 |
| Regulation and Cell signaling | 23 | 25 | 25 | 25 | 27 |
| Secondary Metabolism | 4 | 4 | 4 | 4 | 4 |
| DNA Metabolism | 91 | 96 | 99 | 101 | 107 |
| Fatty acids, Lipids, and Isoprenoids | 75 | 76 | 77 | 77 | 81 |
| Dormancy and sporulation | 1 | 1 | 3 | 3 | 3 |
| Nitrogen Metabolism | 15 | 15 | 15 | 15 | 16 |
| Respiration | 93 | 99 | 96 | 96 | 103 |
| Stress Response | 88 | 92 | 91 | 92 | 97 |
| Metabolism of Aromatic Compounds | 17 | 19 | 35 | 35 | 41 |
| Amino acids and Derivation | 333 | 337 | 350 | 352 | 361 |
| sulfur Metabolism | 32 | 33 | 33 | 33 | 34 |
| Phosphorus Metabolism | 34 | 37 | 34 | 34 | 36 |
| Carbohydrate | 206 | 210 | 241 | 241 | 262 |

**Table S3 Primers used in this study**

| **Names** | **Sequence (5’ to 3’)** |
| --- | --- |
| KVC_1674 (*zwf*)-A | GCGATCTGATAGGAATGC |
| KVC_1674 (*zwf*)-S | TCTTCACATGCTGGATTATG |
| KVC_0890 (*gnd*)-A | CATATTCGATGCCGTTGT |
| KVC_0890 (*gnd*)-S | GTCCGTGTATTGATTGGTT |
| KVC_2247 (*tkt*)-A | GGTAATGCCGTTGTTGTC |
| KVC_2247 (*tkt*)-S | ACCACTATACCTATGTCATCG |
| KVC_1789 (*tal*)-A | GTGACTTCCAGGATGTTG |
| KVC_1789 (*tal*)-S | CGCCGTTATTGAAGAGAT |
| KVC_2082 (*pol*A)-A | GCCAGCAGATTATCCAGA |
| KVC_2082 (*pol*A)-S | CGATCCGATGAAGAACAAG |
